# Extended diagnosis of purine and pyrimidine disorders from urine: LC MS/MS assay development and clinical validation

**DOI:** 10.1371/journal.pone.0212458

**Published:** 2019-02-28

**Authors:** Péter Monostori, Glynis Klinke, Jana Hauke, Sylvia Richter, Jörgen Bierau, Sven F. Garbade, Georg F. Hoffmann, Claus-Dieter Langhans, Dorothea Haas, Jürgen G. Okun

**Affiliations:** 1 Department of General Pediatrics, Division of Neuropediatrics and Metabolic Medicine, Center for Pediatric and Adolescent Medicine, University Hospital Heidelberg, Heidelberg, Germany; 2 Department of Clinical Genetics, Maastricht University Medical Center, Maastricht, The Netherlands; Mayo Clinic Rochester, UNITED STATES

## Abstract

**Background and aims:**

Inborn errors of purine and pyrimidine metabolism are a diverse group of disorders with possible serious or life-threatening symptoms. They may be associated with neurological symptoms, renal stone disease or immunodeficiency. However, the clinical presentation can be nonspecific and mild so that a number of cases may be missed. Previously published assays lacked detection of certain diagnostically important biomarkers, including SAICAr, AICAr, beta-ureidoisobutyric acid, 2,8-dihydroxyadenine and orotidine, necessitating the use of separate assays for their detection. Moreover, the limited sensitivity for some analytes in earlier assays may have hampered the reliable detection of mild cases. Therefore, we aimed to develop a liquid chromatography–tandem mass spectrometry (LC-MS/MS) assay that allows the simultaneous and sensitive detection of an extended range of purine and pyrimidine biomarkers in urine.

**Methods:**

The assay was developed and validated using LC-MS/MS and clinically tested by analyzing ERNDIM Diagnostic Proficiency Testing (DPT) samples and further specimens from patients with various purine and pyrimidine disorders.

**Results:**

Reliable determination of 27 analytes including SAICAr, AICAr, beta-ureidoisobutyric acid, 2,8-dihydroxyadenine and orotidine was achieved in urine following a simple sample preparation. The method clearly distinguished pathological and normal samples and differentiated between purine and pyrimidine defects in all clinical specimens.

**Conclusions:**

A LC-MS/MS assay allowing the simultaneous, sensitive and reliable diagnosis of an extended range of purine and pyrimidine disorders has been developed. The validated method has successfully been tested using ERNDIM Diagnostic Proficiency Testing (DPT) samples and further clinical specimens from patients with various purine and pyrimidine disorders. Sample preparation is simple and assay duration is short, facilitating an easier inclusion of the assay into the diagnostic procedures.

## Introduction

Purine and pyrimidine bases, nucleosides and nucleotides are essential components of the nucleic acids DNA and RNA, and are associated with metabolic regulation, synthesis of numerous biomolecules and other vital processes in cell physiology [1–3] (S1 Fig). Accordingly, inborn errors of purine and pyrimidine metabolism can manifest with serious or life-threatening symptoms that may include immunological, hematological, neurological and renal pathology [1–3].

Purine and pyrimidine deficiencies are generally considered rare; however, their prevalence is unknown and probably underestimated [1, 2]. One reason is that their clinical spectrum is diverse and frequently overlapping with other disorders. Moreover, there is considerable heterogeneity in the phenotype and concentrations of biomarkers even within an affected family. Third, although previously considered pediatric diseases, these disorders are now increasingly being diagnosed in adults as milder presentations and mutations are recognized [1, 2]. This underlines the importance of analytical assays that can detect biomarkers of purine and pyrimidine metabolism with sufficient sensitivity and specificity [1, 2]. For clinical diagnosis, liquid chromatography–tandem mass spectrometry (LC-MS/MS) is the technique of choice [4], predominantly using urine as sample matrix [1, 2].

Despite the availability of clinical LC-MS/MS methods for purines and pyrimidines [5–9], there is still significant need for improvement. First, previously published assays lacked detection of certain diagnostically important biomarkers, including SAICAr (succinyl-5-aminoimidazole-4-carboxamide-1-ribonucleoside; a marker of adenylosuccinate lyase deficiency, ADSL), not included in [5–9]; AICAr (5-aminoimidazole-4-carboxamide ribonucleoside; a marker of AICAR transformylase/IMP cyclohydrolase deficiency, ATIC), not assayed in [5–9]; beta-ureidoisobutyric acid (beta-ureidopropionase deficiency, UPB1), not detected in [5, 7, 9]; 2,8-dihydroxyadenine (adenine phosphoribosyltransferase deficiency, APRT), not included in [5, 7–9] and orotidine (uridine monophosphate synthetase deficiency (UMPS) type III), not assayed in [5–9]. Thus, in order to avoid missing cases, separate assays such as the Bratton-Marshall-test for SAICAr [10] were recommended to be performed additionally for detection of these additional metabolites. In addition, some analytes could only be measured with limited sensitivity in those earlier LC-MS/MS assays [5–9], potentially preventing reliable detection of milder disease forms.

Therefore, we aimed to develop an LC-MS/MS assay that allows the reliable diagnosis of an extended range of purine and pyrimidine disorders while still maintaining sample preparation simple and assay duration short for easier inclusion of the assay in the routine workflow. To verify the clinical performance of the assay, we set out to analyze ERNDIM Diagnostic Proficiency Testing (DPT) samples and further specimens from known patients.

## Materials and methods

### Reagents

Unlabeled standards were purchased from Sigma-Aldrich (St. Louis, MO, USA), except for 2,8-dihydroxyadenine, dihydrothymine, orotidine and succinyladenosine (Toronto Research Chemicals, Toronto, ON, Canada) and pseudouridine (Berry & Associates, Dexter, MI, USA). SAICAr was obtained from Dr. Marie Zikánová (Charles University, Prague, Czech Republic). Deuterated internal standards (ISs) were acquired from Cambridge Isotope Laboratories (Andover, MA, USA). Methanol, isopropanol and formic acid (all ULC-MS grade) were purchased from Biosolve (Valkenswaard, The Netherlands). Ultrapure water (18.2 MΩ.cm), filtered through a 0.22 μm pore size membrane, was obtained from a Thermo Barnstead GenPure Pro UV-TOC/UF system (Thermo Fisher Scientific, Waltham, MA, USA).

### Calibrators and quality controls

The analyte levels in calibrators Cal01 to Cal10 prior to sample preparation were as follows: 0, 1, 2, 4, 8, 16, 32, 64, 128 and 256 μM, respectively. During method validation and in subsequent analyses, we used the „Control Purines and Pyrimidines” material from ERNDIM (supplier: MCA Laboratory, Queen Beatrix Hospital, Winterswijk, The Netherlands) as an officially approved external quality control (EQC; the LOT 2015.010 was used for method development). In order to monitor further analytes not included in the EQC, we also prepared an internal QC (IQC) in-house that contained all metabolites of the EQC plus beta-ureidopropionic acid, allopurinol, beta-ureidoisobutyric acid, 2,8-dihydroxyadenine and succinyladenosine. Since analyte levels in the EQC represented the highly pathologic range, concentrations in the IQC were chosen to be lower but still analytically relevant, i.e. 20 μM. All calibrators, the IQC and EQC samples and the IS working solution were stored in aliquots at -20°C. The preparation of standard stock solutions, calibrators, IS stock and working solutions and QCs are further detailed in the S1 Text.

### Sample preparation

Urinary creatinine concentrations were determined with a Beckman Coulter AU480 Chemistry System, using a kinetic modification of the Jaffe procedure (Beckman Coulter Diagnostics, Brea, CA, USA). Urine samples were centrifuged for 5 min at 400 × *g* in a Hettich Rotina 420R centrifuge (Tuttlingen, Germany) at ambient temperature. Sample preparation was performed in a 96-well Merck MultiScreen filter plate (pore size: 0.45 μm, volume: 2 ml), with a Merck MultiScreen collection plate (volume: 2 ml). In order to obtain creatinine levels of 0.25 mM in all samples at the first stage of sample preparation, appropriate volumes of urine samples (based on creatinine levels) were diluted to 400 μl with water, whereas 100 μl aliquots of the calibrators and QCs were mixed with 300 μl synthetic urine (creatinine 0.333 mM, Synthetic Urine e. K., Nußdorf, Germany). Then, 100 μl 25 μM IS was added to all samples. Accordingly, the final concentrations of creatinine and IS in the samples were 0.20 mM and 5 μM, respectively, with a final sample volume of 500 μl.

The samples were sealed with an adhesive foil (ChromSystems, Gräfelfing, Germany), mixed and incubated for 10 min at ambient temperature. Then, the samples were filtered using a 96-well Plate Vacuum Manifold (Phenomenex, Torrance, CA, USA). The collection plate was sealed with a pre-slit adhesive foil (ChromSystems, Gräfelfing, Germany) and placed directly into the autosampler. Results of the LC-MS/MS analysis were reported in mmol/mol creatinine.

### LC-MS/MS instruments and settings

A Waters Acquity I-Class UPLC system (Binary Solvent Manager, thermostatic Column Manager and FTN Sample Manager) and a Waters TQ-S triple quadrupole MS/MS were used, controlled by MassLynx 4.1 software (all Waters, Milford, MA, USA). MS/MS data were evaluated with TargetLynx 4.1 software (Waters, Milford, MA, USA).

The chromatographic separation of the analytes was performed on an ACE Excel C18-AR 100×3.0mm; 1.7 μm analytical column (Advanced Chromatography Technologies, Aberdeen, Scotland). Eluent A consisted of ultrapure water plus 0.4% formic acid (ULC-MS grade). Eluent B consisted of methanol/water 50/50 (methanol ULC-MS grade). Both eluents were prepared weekly. The column was tempered to 25°C. The autosampler settings were the following: injection volume: 1 μl; sample compartment temperature: 5°C. Gradient elution at a flow of 200 μl/min was performed by changing %B as follows: 0.0-1.1 min: 10%; 1.1-6.0 min: 10% to 80%; 6.0-6.1 min: 80% to 100%; 6.1-8.0 min: 100%; 8.0-8.5 min: 100% to 10%; 8.5-14.0 min: 10%. The flow was directed to waste in the first 2 min and the last 5.5 min of each acquisition. Further experience with columns, eluents and centrifugal filter cups obtained during method development are provided in the S1 Table.

MS/MS settings were optimized by means of consecutive syringe infusions of 1 μM solutions for each analyte and IS, dissolved in methanol/water 50/50. Optimized MS/MS settings, including electrospray ion (ESI) modes of purines and pyrimidines and related metabolites are summarized in Tables 1 and 2, respectively. In the final MS/MS method, all MRMs were acquired within 3 min acquisition windows (retention time ±1.5 min), with dwell times of 5 ms for each MRM. The final ion source settings were the following: desolvation gas flow = 800 L/h; cone gas flow = 150 L/h; nebulizer = 6.0 bar; capillary voltage = 2.5 kV; desolvation temperature = 650°C; source temperature = 150°C.

**Table 1:** Optimized MS/MS settings of purines and related metabolites.

Analyte	Internal standard	ESI mode	Quantifier/ Qualifier	Parent mass (Da)	Daughter mass (Da)	Cone voltage (V)	Collision voltage (V)
Adenine	Xanthine-IS (1,3-^15^N_2_)	Positive	QUANT	136.0	92.0	66	24
		Positive	qual	136.0	119.1	66	20
Hypoxanthine	Hypoxanthine-IS (^13^C_5_)	Positive	QUANT	137.1	110.1	42	18
		Positive	qual	137.1	81.9	42	18
Allopurinol	Hypoxanthine-IS (^13^C_5_)	Positive	QUANT	137.1	54.1	56	22
		Positive	qual	137.1	110.0	56	18
Hypoxanthine-IS (^13^C_5_)	n.a.	Positive	QUANT	142.0	114.1	40	20
		Positive	qual	142.0	124.1	40	20
Xanthine	Xanthine-IS (1,3-^15^N_2_)	Positive	QUANT	153.1	110.1	26	18
		Positive	qual	153.1	136.0	26	12
Xanthine-IS (1,3-^15^N_2_)	n.a.	Positive	QUANT	155.1	137.1	20	14
		Positive	qual	–	–	–	–
2,8-Dihydroxyadenine	Xanthine-IS (1,3-^15^N_2_)	Positive	QUANT	168.1	125.0	66	18
		Positive	qual	168.1	150.8	66	18
Deoxyadenosine	Guanosine-IS (^15^N_5_)	Positive	QUANT	252.1	119.0	18	38
		Positive	qual	252.1	136.1	18	16
AICAr	Guanosine-IS (^15^N_5_)	Positive	QUANT	259.2	110.1	26	22
		Positive	qual	259.2	127.1	26	10
Adenosine	Guanosine-IS (^15^N_5_)	Positive	QUANT	268.2	119.0	30	44
		Positive	qual	268.2	136.1	30	18
Guanosine-IS (^15^N_5_) ESI+	n.a.	Positive	QUANT	289.1	139.1	20	40
		Positive	qual	289.1	157.1	20	14
Deoxyinosine	Guanosine-IS (^15^N_5_)	Negative	QUANT	251.1	135.1	56	22
		Negative	qual	251.1	161.1	56	24
Deoxyguanosine	Deoxyguanosine-IS (^13^C_10_^15^N_5_)	Negative	QUANT	266.1	150.0	54	20
		Negative	qual	266.1	133.1	54	28
Inosine	Inosine-IS (^15^N_4_)	Negative	QUANT	267.1	135.1	58	20
		Negative	qual	267.1	92.0	58	34
Inosine-IS (^15^N_4_)	n.a.	Negative	QUANT	271.1	139.1	20	20
		Negative	qual	–	–	–	–
Deoxyguanosine-IS (^13^C_10_^15^N_5_)	n.a.	Negative	QUANT	281.1	160.1	20	20
		Negative	qual	281.1	142.1	20	30
Guanosine	Guanosine-IS (^15^N_5_)	Negative	QUANT	282.1	150.0	48	18
		Negative	qual	282.1	133.1	48	30
Guanosine-IS (^15^N_5_) ESI–	n.a.	Negative	QUANT	287.1	155.1	20	18
		Negative	qual	287.1	137.1	20	30
SAICAr	Guanosine-IS (^15^N_5_)	Negative	QUANT	373.1	355.1	20	18
		Negative	qual	373.1	294.1	20	20
Succinyladenosine	Guanosine-IS (^15^N_5_)	Negative	QUANT	382.1	206.2	40	20
		Negative	qual	382.1	134.1	40	30

ESI: electrospray ionization; IS: internal standard; AICAr: 5-aminoimidazole-4-carboxamide ribonucleoside; SAICAr: succinyl-5-aminoimidazole-4-carboxamide-1-ribonucleoside; n.a.: not applicable. Note that guanosine-IS is measured in both ESI+ and ESI-.

**Table 2:** Optimized MS/MS settings of pyrimidines and related metabolites.

Analyte	Internal standard	ESI mode	Quantifier/ Qualifier	Parent mass (Da)	Daughter mass (Da)	Cone voltage (V)	Collision voltage (V)
Uracil	Uracil-IS (1,3-^15^N_2_)	Positive	QUANT	113.0	70.0	50	14
		Positive	qual	113.0	96.1	50	14
Dihydrouracil	Dihydrouracil-IS (^13^C_4_^15^N_2_)	Positive	QUANT	115.0	55.0	40	15
		Positive	qual	115.0	73.0	40	10
Uracil-IS (1,3-^15^N_2_)	n.a.	Positive	QUANT	115.1	71.0	30	14
		Positive	qual	115.1	97.1	30	14
Dihydrouracil-IS (^13^C_4_^15^N_2_)	n.a.	Positive	QUANT	121.0	58.0	40	16
		Positive	qual	121.0	77.0	40	12
Thymine	Thymine-IS (D_4_)	Positive	QUANT	127.0	110.0	50	16
		Positive	qual	127.0	84.1	50	14
Dihydrothymine	Dihydrothymine-IS (D_3_ + methyl-D_3_)	Positive	QUANT	129.0	112.0	34	10
		Positive	qual	129.0	69.0	34	14
Thymine-IS (D_4_)	n.a.	Positive	QUANT	131.1	114.1	40	15
		Positive	qual	131.1	88.1	40	16
Beta-Ureidopropionic acid	Uracil-IS (1,3-^15^N_2_)	Positive	QUANT	133.0	90.1	20	8
		Positive	qual	133.0	72.2	20	12
Dihydrothymine-IS (D_3_ + methyl-D_3_)	n.a.	Positive	QUANT	135.1	74.1	30	14
		Positive	qual	135.1	90.1	30	13
Beta-Ureidoisobutyric acid	Uracil-IS (1,3-^15^N_2_)	Positive	QUANT	147.0	86.0	24	14
		Positive	qual	147.0	129.0	24	8
5-Hydroxymethyluracil	Orotic Acid-IS (1,3-^15^N_2_)	Negative	QUANT	141.0	123.1	42	12
		Negative	qual	141.0	42.0	42	14
Orotic acid	Orotic Acid-IS (1,3-^15^N_2_)	Negative	QUANT	155.0	111.0	34	10
		Negative	qual	155.0	42.0	34	20
Orotic Acid-IS (1,3-^15^N_2_)	n.a.	Negative	QUANT	157.1	113.1	30	10
		Negative	qual	157.1	43.1	30	20
Deoxyuridine	Guanosine-IS (^15^N_5_)	Negative	QUANT	227.0	184.1	44	12
		Negative	qual	227.0	94.1	44	22
Thymidine	Thymidine-IS (^13^C_10_^15^N_2_)	Negative	QUANT	241.1	42.0	44	12
		Negative	qual	241.1	151.1	44	10
Pseudouridine	Orotic Acid-IS (1,3-^15^N_2_)	Negative	QUANT	243.0	153.1	36	12
		Negative	qual	243.0	183.1	36	14
Thymidine-IS (^13^C_10_^15^N_2_)	n.a.	Negative	QUANT	253.1	160.1	30	10
		Negative	qual	253.1	44.1	30	12
Orotidine	Orotic Acid-IS (1,3-^15^N_2_)	Negative	QUANT	287.1	111.1	20	15
		Negative	qual	287.1	42.1	20	22

ESI: electrospray ionization; IS: internal standard; n.a.: not applicable.

### Application of the LC-MS/MS assay on ERNDIM Diagnostic Proficiency Testing (DPT) samples and further patient specimens

Diagnostic testing of the LC-MS/MS assay was first performed by analyzing the levels of purines and pyrimidines in ERNDIM Diagnostic Proficiency Testing (DPT) samples (*n* = 10). Subsequently, further urine specimens from patients (*n* = 10) referred for routine purine-pyrimidine determination using a previously published method [6] were re-assayed with the present method. All procedures followed were in accordance with the ethical standards of the Helsinki Declaration of 1975, as revised in 2000 and approved by the Ethical Committee of the University of Heidelberg (071/2005). Written consent was obtained from ERNDIM as an approval to use the DPT samples in method development.

## Results

### Method validation

The linearity (coefficient of determination, R^2^), the limit of detection (LOD) and the lower and upper limits of quantitation (LLOQ, ULOQ) were tested after serial dilution and subsequent sample preparation of calibrators (*n* = 10) (S2 Table). LOD was defined as S/N = 3; LLOQ was defined as the lowest concentration measured with a coefficient of variation (CV) lower than 20%; ULOQ was defined as the upper end of the range where the response of a given analyte was linear, i.e. where the coefficient of determination (R^2^) was higher than 0.99.

The chromatographic resolution (*R*_*s*_) of critical analyte pairs showing MS/MS interference was calculated via the following equation: *R*_*s*_ *= 1*.*18(t*_*2*_*-t*_*1*_*)/(w*_*0*.*5*,*1*_*+w*_*0*.*5*,*2*_*)* where *t*_*1*_ and *t*_*2*_ are the retention times of the respective peaks and *w*_*0*.*5*,*1*_ and *w*_*0*.*5*,*2*_ are the peak widths at half peak height [11]. Baseline resolution (*R*_*s*_≥1.5) was achieved for all critical pairs (S2 Table).

Carryover was tested by injecting blank samples (methanol) after the highest calibrator Cal10. No peaks were detected in the blank samples.

Interday and intraday precisions, variations between multiple injections of a single sample extract and recoveries were examined using both the IQC and the EQC samples (*n* = 10) (S3 Table and Table 3, respectively). To highlight the reliability of the method, the interday reproducibility data were calculated and reported from 10 measurements on 10 consecutive weeks, not 10 consecutive days. Note, that the IQC sample included additional analytes, as compared with the ERNDIM EQC sample.

**Table 3:** Assay validation using the ERNDIM „Control Purines and Pyrimidines” external quality control samples (EQC; LOT used for method development: 2015.010) (*n* = 10).

Analyte	Analyte level (μM)	CV (%) interday	CV (%) intraday	CV (%) between injections of a single sample extract	Recovery (%)
Uracil	71.8	3.7	3.0	4.1	105.1
Dihydrouracil	65.1	6.2	4.4	3.6	118.4
Thymine	39.5	3.4	2.5	4.3	107.7
Dihydrothymine	70.5	3.9	3.4	2.7	108.7
Beta-Ureidopropionic acid*	n.a.	n.a.	n.a.	n.a.	n.a.
Adenine	28.0	4.8	2.8	2.6	123.8
Hypoxanthine	116.0	4.6	2.9	3.2	94.5
Allopurinol*	n.a.	n.a.	n.a.	n.a.	n.a.
Beta-Ureidoisobutyric acid*	n.a.	n.a.	n.a.	n.a.	n.a.
Xanthine	95.9	3.9	3.5	3.6	107.1
2,8-Dihydroxyadenine*	n.a.	n.a.	n.a.	n.a.	n.a.
Deoxyadenosine	38.0	4.7	4.3	4.3	119.7
AICAr	52.5	5.7	4.0	1.9	94.1
Adenosine	34.8	6.3	4.6	2.7	110.6
5-Hydroxymethyluracil	49.7	8.5	5.9	6.9	103.4
Orotic acid	80.0	4.1	2.3	3.6	97.8
Deoxyuridine	43.1	8.6	3.9	3.2	92.8
Thymidine	22.8	8.8	11.6	9.7	89.4
Pseudouridine	90.3	6.3	2.2	3.1	108.4
Deoxyinosine	38.4	2.6	1.3	3.0	101.3
Deoxyguanosine	37.9	3.0	4.4	3.1	100.5
Inosine	50.0	3.5	2.9	2.8	97.0
Guanosine	56.4	3.4	2.8	2.0	95.7
Orotidine	14.0	5.9	5.4	4.3	82.9
Succinyladenosine*	n.a.	n.a.	n.a.	n.a.	n.a.

* Analyte not included in the ERNDIM EQC sample.

CV: coefficient of variation; AICAr: 5-aminoimidazole-4-carboxamide ribonucleoside; n.a.: not applicable.

Unprepared calibrators and QCs were stable with CV<15% for all analytes for at least 6 months if stored aliquoted at -20°C.

The stability of the prepared samples during storage was evaluated by aliquoting a set of calibrators and QCs immediately after sample preparation, with one aliquot measured both immediately and following 48h of storage at 5°C in the autosampler and another aliquot assayed after 7 days of storage at -20°C. There were no significant differences in the peak intensities, the linear curve equations or the calculated concentrations of the QCs.

### Reference intervals

For determination of normal biomarker ranges, urine samples (*n* = 251) referred for routine purine-pyrimidine measurements using a previously published method [6] were re-assayed with the present method. The specimens (collected between 0-18 years of age) were randomly chosen and anonymized prior to the analysis. Reference intervals and cutoffs were determined according to the following statistical procedure.

The R environment for statistical computing and graphics was used to analyze data [12]. Initially, for each metabolite, values above 99.5^th^ percentile were excluded. The 97.5^th^ percentile of the remaining data defined the upper cutoff of an analyte. For pseudouridine, a lower cutoff was also defined as the 2.5^th^ percentile. The possible age dependency of the cutoffs was tested as follows. A one-way analysis of variance (ANOVA) based on the 97.5^th^ percentile and, additionally for pseudouridine, on the 2.5^th^ percentile was computed [13] to test if the cutoffs differed significantly between the age groups 0-1, 1-3, 3-6 and 6-18 years, respectively. Where quantile ANOVA detected significantly different 97.5^th^ (or 2.5^th^) percentiles between certain age groups and metabolic specialists also rated the difference as clinically relevant, age-related cutoffs were defined for the respective groups. The cutoff values are provided in Table 4.

**Table 4:** Cutoff values of the measured analytes.

Analyte	Cutoff (μM)
Uracil	30.0
Dihydrouracil	0-1y: 34.0; >1y: 12.0
Thymine	1.2
Dihydrothymine	6.0
Beta-Ureidopropionic acid	0-1y: 32.0; >1y: 12.0
Adenine	1.0
Hypoxanthine	52.0
Allopurinol	n.d.
Beta-Ureidoisobutyric acid	0-1y: 9.0; >1y: 2.5
Xanthine	0-1y: 43.0; >1y: 31.0
2,8-Dihydroxyadenine	4.0
Deoxyadenosine	1.0
AICAr	1.1
Adenosine	2.0
5-Hydroxymethyluracil	1.0
Orotic acid	4.0
Deoxyuridine	3.0
Thymidine	1.0
Pseudouridine	0-1y: 37.0–110.0; >1y: 30.0–110.0
Deoxyinosine	1.0
Deoxyguanosine	1.0
Inosine	0-3y: 5.0; >3y: 2.0
Guanosine	1.0
Orotidine	4.7
SAICAr	0.8
Succinyladenosine	5.0

AICAr: 5-aminoimidazole-4-carboxamide ribonucleoside; SAICAr: succinyl-5-aminoimidazole-4-carboxamide-1-ribonucleoside.

### Testing of the present assay with ERNDIM Diagnostic Proficiency Testing (DPT) samples and further clinical specimens

**Fig 1:**
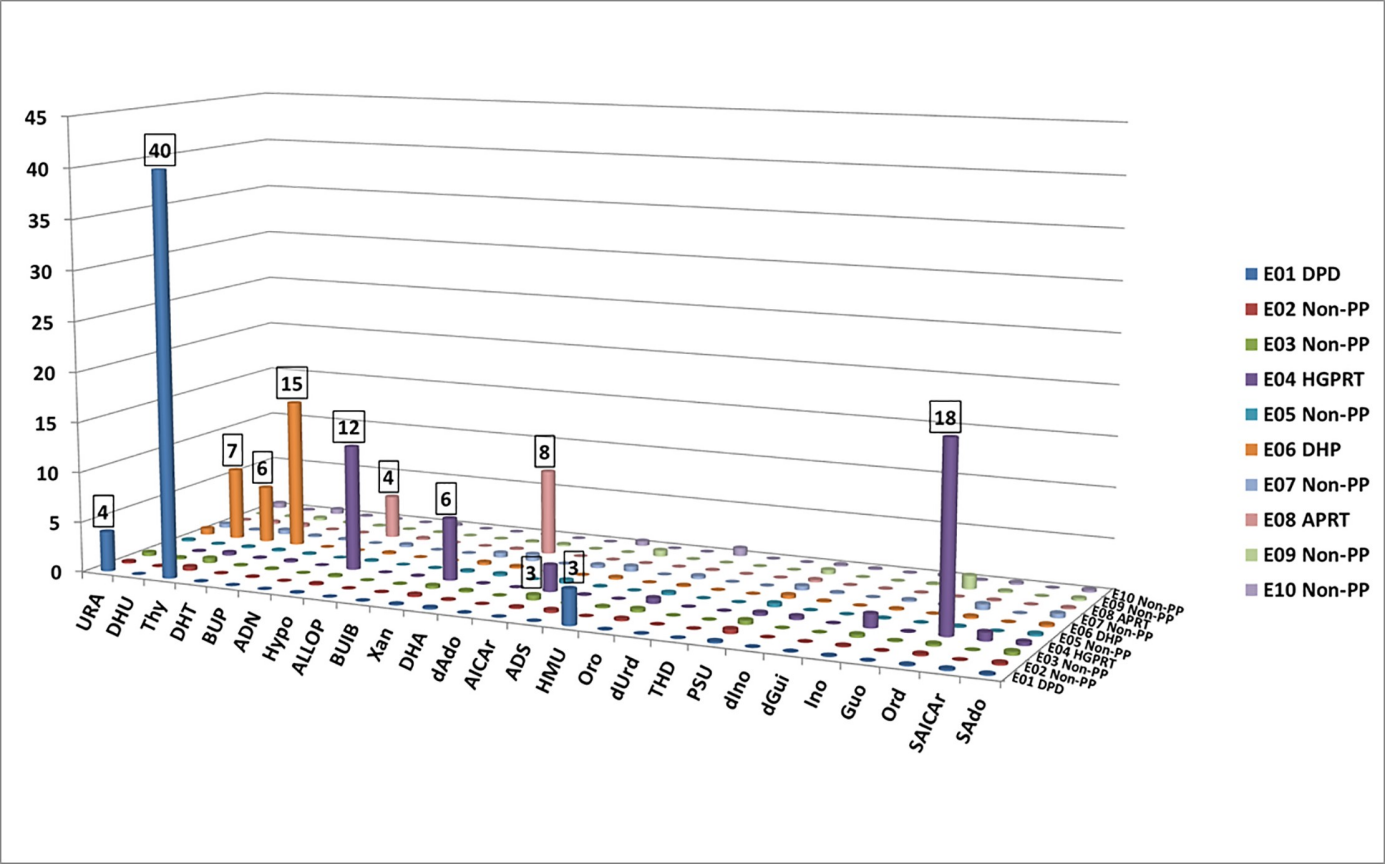
Results of the ERNDIM Diagnostic Proficiency Testing (DPT) samples (*n* = 10). **Numbers on the cylinders show how many times a given result exceeded the respective cutoff for that biomarker.** PP: purine-pyrimidine disorder. Further abbreviations are provided in the List of Abbreviations Section. Note that orotidine is not pathognomonic for HGPRT (sample E04) but is accumulated as a result of allopurinol treatment.

The levels of purines and pyrimidines were measured in ERNDIM Diagnostic Proficiency Testing (DPT) samples (*n* = 10). The new assay clearly identified the pathological and the normal metabolite patterns and correctly differentiated between purine-pyrimidine disorders in all cases (Fig 1).

**Fig 2:**
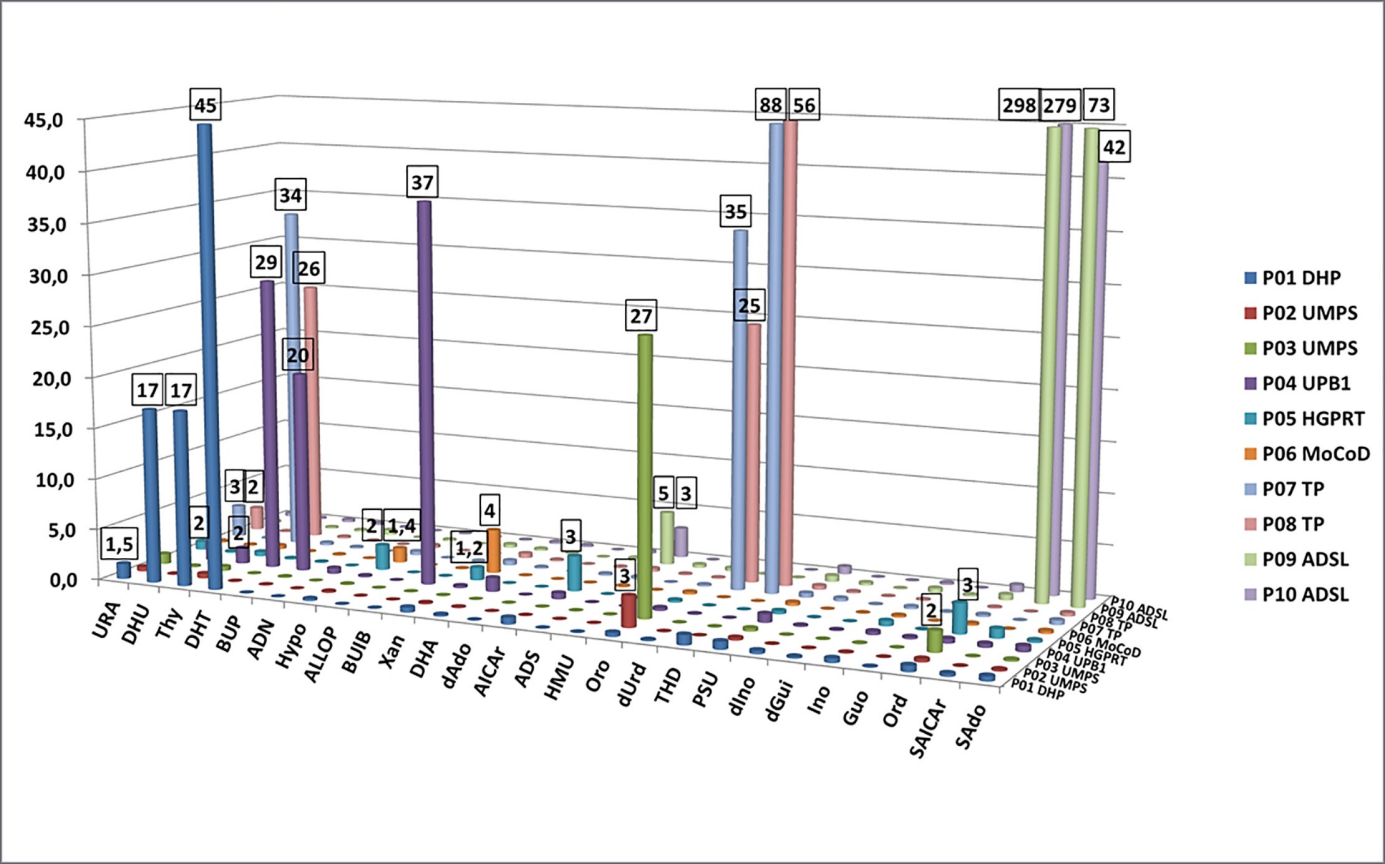
Results of urine samples from patients with known diagnosis (*n* = 10). Numbers on the cylinders show how many times a given result exceeded the respective cutoff for that biomarker. Abbreviations are provided in the List of Abbreviations Section. The Y-axis has been cut at 45 for a better visibility. Note that orotidine is not pathognomonic for HGPRT (sample P05) but is accumulated as a result of allopurinol treatment.

Results of the analyses of further patient specimens (*n* = 10) are depicted in Fig 2. The new LC-MS/MS assay clearly distinguished the pathological samples from normal ones and correctly diagnosed purine-pyrimidine disorders in all tested specimens. Selected clinical data of the patients are given in S2 Text.

To investigate the possible effects of urine pH on the LC-MS/MS results, we tested urine samples with very low creatinine levels, which allowed testing the highest possible amount of urine in the sample preparation. Three aliquots were prepared in each of two urine samples. 20 μl of 0.1M formic acid, 0.1M NaOH or water was added to the aliquots, respectively, resulting in a ±1 unit shift of the original urine pH (originally 6 and 7 for the two urine samples, respectively). There were no differences between the calculated concentrations of the three corresponding aliquots.

**Fig 3:**
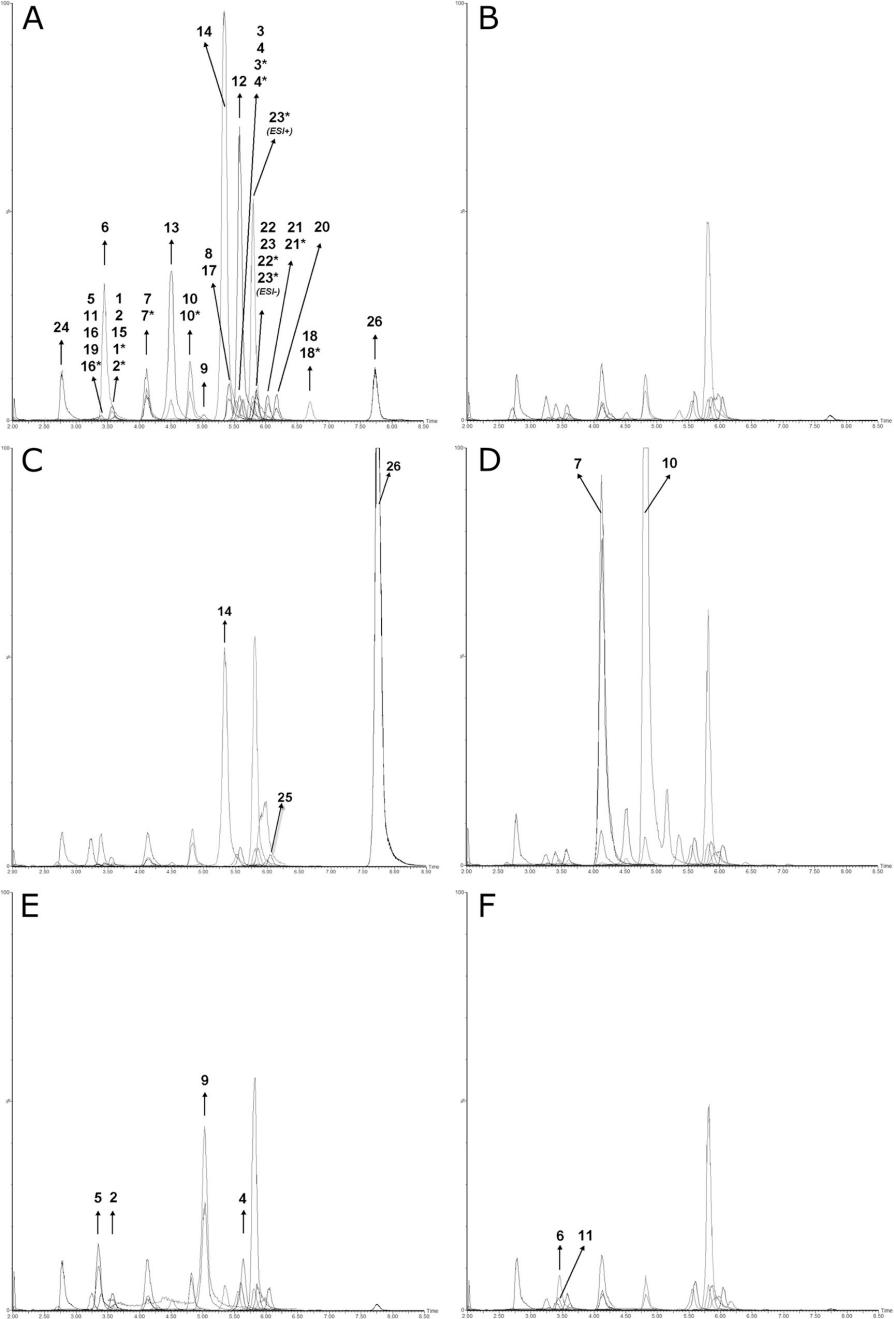
**LC-MS/MS chromatograms of A: the Cal06 calibrator (16 μM) and urine specimens from B: a healthy proband; C: a patient with ADSL deficiency; D: a patient with HGPRT deficiency; E: a patient with UPB1 deficiency; and F: a patient with APRT deficiency.** All intensities (Y-axes) have been normalized to 1.5*10^8^ cps for better comparability and have been plotted against retention time (X-axes). Analytes have been numbered as follows: 1. URA; 2. DHU; 3. Thy; 4. DHT; 5. BUP; 6. ADN; 7. Hypo; 8. ALLOP; 9. BUIB; 10. Xan; 11. DHA; 12. dAdo; 13. AICAr; 14. ADS; 15. HMU; 16. Oro; 17. dUrd; 18. THD; 19. PSU; 20. dIno; 21. dGui; 22. Ino; 23. Guo; 24. Ord; 25. SAICAr; 26. SAdo. The ISs have been marked by an asterisk (*) after the number of the respective unlabeled analyte. Note that guanosine-IS has been measured in both ESI+ and ESI-. ADSL: Adenylosuccinate lyase deficiency; HGPRT: Hypoxanthine-guanine phosphoribosyltransferase deficiency; UPB1: beta-Ureidopropionase deficiency; APRT: Adenine phosphoribosyltransferase deficiency. Further abbreviations are provided in the List of Abbreviations Section. Chromatograms were generated by MassLynx 4.1 (Waters, Milford, MA, USA) and modified using Inkscape 0.91pre4 (Open Source Software licensed under the GNU General Public License).

Representative LC-MS/MS chromatograms of a calibrator, a healthy proband and patients with ADSL, HGPRT, UPB1 and APRT deficiencies, respectively, are presented in Fig 3.

## Discussion

Inborn errors of purine and pyrimidine metabolism are characterized by a broad phenotypic spectrum, in line with the ubiquitous presence and biochemical importance of these metabolites [1–3]. Clinically, purine and pyrimidine deficiencies can present with serious immunological, hematological, neurological and renal symptoms including delayed development, epilepsy, muscular hyper- or hypotonicity, mental retardation, dysmorphic features, neurogastrointestinal symptoms, ophthalmoplegia, muscle atrophy and polyneuropathy [1–3]. However, the clinical presentation can be nonspecific and mild which may result in missing the diagnosis [1, 2].

Nucleotides within cells break down to form the respective nucleosides and bases, which are transported into the extracellular fluids [1]. Thus, the diagnosis of purine and pyrimidine disorders is generally performed by detecting the related nucleosides, bases or degradation products associated with the enzyme defect. Urinary excretion profiles are most commonly used but application of plasma, serum or cerebrospinal fluid have also been published [1, 2]. Only very few disorders were reported to be detectable from dried blood spots which could possibly allow their inclusion in newborn screening [14–16].

Traditionally, common methods for the detection of purine and pyrimidine metabolites applied HPLC combined with diode array or UV detection or, for pyrimidine degradation products, GC-MS. ^1^H-NMR was also reported to be appropriate for the diagnosis of many but not all of these disorders [17], still the most applied technique currently is LC-MS/MS [4].

To the best of our knowledge, the LC-MS/MS assay developed and presented here is the first one published to include the biomarkers SAICAr, AICAr, beta-ureidoisobutyric acid, 2,8-dihydroxyadenine and orotidine in a single method, in addition to the previously reported panel of purines and pyrimidines. This extension is expected to facilitate the detection of the disorders ADSL [1, 3], ATIC [1, 3], UPB1 [2, 3], APRT [1, 3] and UMPS [2, 18, 19], respectively, together with the deficiencies of HGPRT [1, 3], molybdenum cofactor (MoCoD) [1, 3], thymidine phosphorylase (TP) [2, 3], dihydropyrimidine dehydrogenase (DPD) [2, 3] and dihydropyrimidinase (DHP) [2, 3]. Based on the analyte panel, the presented assay should additionally be able to detect deficiencies of adenosine deaminase (ADA) [1, 3], purine nucleoside phosphorylase (PNP) [1, 3] and xanthine dehydrogenase (XDH) [1, 3], as well as phosphoribosylpyrophosphate synthetase (PRPPS) superactivity [1, 3]. However, this should be confirmed using patient samples with these disorders, which were not available for the authors of the present paper.

Many previously published LC-MS/MS methods aimed to assay several metabolites simultaneously in order to be able to diagnose multiple disorders [5–9] but all of them lacked detection of one or other of the above biomarkers. Even in assays using emerging techniques for the detection of a broad range of analytes, such as LC-QTOF [20–22], the number of currently diagnosable purine and pyrimidine disorders was substantially lower than in the present assay. As an example, the detection of SAICAr, a marker of ADSL deficiency, is commonly performed using the Bratton-Marshall-test [10]. However, this color reaction test is neither quantitative nor specific as it may give positive results with a number of structurally related purine intermediates [10]. Previously, only few assays were reported for the quantitative measurement of SAICAr [14; 23; 24] which, in turn, allowed the detection of a much lower number of additional analytes.

To improve the quantitation of purines and pyrimidines, we have used a large number of isotopically labeled ISs (total: 11) as compared with earlier reports [5, 6, 8, 9]. The use of multiple ISs has not only improved the reliability of quantitation for analytes having an own labeled analog, but also allowed us to select the most appropriate IS for those analytes where a labeled analog was not commercially available. The selection was based on numerous factors, including the long-term stability of calibration, linearity (absence of saturation) and similarities in structure and retention time [25, 26].

In line with the above approach, we have decided to omit the labeled analogs of adenine and adenosine following extensive testing, despite being commercially available. The exclusion of labeled adenine-IS and adenosine-IS eliminated crosstalk between these single labeled ISs and their unlabeled counterparts, having mass differences of only one Da [25] and also helped to decrease ion suppression for high analyte levels [26]. Instead, xanthine-IS and guanosine-IS, respectively, were found to perform well as ISs, exhibiting an improved stability of calibration and a wider linear range.

Note that even if 2,8-dihydroxyadenine had been included in the calibrators during assay validation, it was omitted from the standard row later. The variable responses of 2,8-dihydroxyadenine, observable exclusively among distinct charges of calibrators, were presumably related to the very poor solubility of 2,8-dihydroxyadenine [1]. In turn, the responses of 2,8-dihydroxyadenine in urine samples were stable under the current assay conditions. Thus, this issue could have been resolved by using the same calibration settings obtained during validation to quantify 2,8-dihydroxyadenine in subsequent sample preparations. In accordance, we could correctly identify the ERNDIM Diagnostic Proficiency Testing (DPT) sample E08 having elevated adenine and 2,8-dihydroxyadenine levels, characteristic for APRT deficiency (Fig 2). The same approach was used for the quantitation of SAICAr due to difficulties in the availability of the standard substance. In line, two patient specimens with ADSL deficiency (P09 and P10) were correctly identified (S3 Table).

Another limitation of the present assay is that the inclusion and quantitation of uric acid, the end-product of purine degradation, was not possible under the current sample preparation and LC conditions. In addition to the low temperature in the autosampler, otherwise favorable in terms of stability for most analytes, a low pH was also associated with a decreased solubility of uric acid [27]. On the other hand, acidic conditions were shown to be important not only for the adequate chromatographic separation of the analytes in the present assay but were also critical for the stability of dihydrouracil and dihydrothymine which would have been degraded to beta-ureidopropionic acid and beta-ureidoisobutyric acid under higher pH (see also S1 Text) [28]. Since uric acid can routinely be measured using traditional techniques such as colorimetric or enzymatic assays, we opted to maintain the pH in the acidic range throughout sample preparation and measurement in favor of all other analytes.

The use of a well-chosen column and dedicated method development allowed us to achieve ultra-high performance in terms of sensitivity, resolution and clinical reliability and to still remain in pressure ranges close to but not exceeding the limits of conventional high-performance liquid chromatography (HPLC). This approach, together with simple sample preparation, is expected to facilitate an easier inclusion of this assay in the daily routine and to improve transferability among laboratories. The ongoing prospective evaluation of the presented method in our laboratory and a wider range of patient samples can provide further experience on the reliability of the assay to identify patients in the routine setting.

## Conclusions

An LC-MS/MS assay allowing the simultaneous, sensitive and reliable diagnosis of an extended range of purine and pyrimidine disorders has been developed. The validated assay has been tested using ERNDIM Diagnostic Proficiency Testing (DPT) samples and further clinical specimens from patients with various purine and pyrimidine disorders. The method clearly distinguished the pathological and normal samples and differentiated between purine and pyrimidine defects in all tested specimens. Sample preparation is simple and assay duration is short, facilitating an easier inclusion of the method in a routine workflow. The prospective evaluation of the described method is currently being continued in our laboratory.

## Supporting information

S1 Fig Biochemical pathways of purine and pyrimidine metabolism (simplified). **Metabolites detected by the presented method are printed in bold**.
Purines:
*Enzymes of the de novo purine synthesis*: PRPPS: phosphoribosyl pyrophosphate synthetase; ADSL: adenylosuccinate lyase (adenylosuccinase); ATIC: AICAR transformylase/IMP cyclohydrolase; ADSS: adenylosuccinate synthetase. *Enzymes of purine catabolism*: AMPA: AMP deaminase; 5NT: 5'-nucleotidase(s); ADA: adenosine deaminase; PNP: purine nucleoside phosphorylase; XDH: xanthine dehydrogenase (xanthine oxidase); GDA: guanine deaminase; IMPDH: IMP dehydrogenase. *Enzymes of purine salvage*: HGPRT: hypoxanthine-guanine phosphoribosyltransferase; dGUOK: deoxyguanosine kinase; APRT: adenine phosphoribosyltransferase; ADK: adenosine kinase.
Pyrimidines:
*Enzymes of the de novo pyrimidine synthesis*: CPS2: carbamoylphosphate synthetase II: ATC: aspartate transcarbamoylase; DHO: dihydroorotase (CAD is comprised of CPS2, ATC and DHO); DHODH: dihydroorotate dehydrogenase; OPRT: orotate phosphoribosyltransferase; OMPDC: OMP decarboxylase (UMPS = uridine monophosphate synthetase is comprised of OPRT and OMPDC). *Enzymes of pyrimidine catabolism*: 5NT: 5'-nucleotidase(s); UP: uridine phosphorylase(s); TP: thymidine phosphorylase; DPD: dihydropyrimidine dehydrogenase; DHP: dihydropyrimidinase; UPB1: beta-ureidopropionase. *Enzymes of pyrimidine salvage*: PUS: pseudouridine synthase; TDO: thymine dioxygenase. Ribonucleotide reductase (RNR) and thymidylate synthetase (TYMS) are used in the synthesis of deoxynucleotides.
AMP: adenosine-5’-monophosphate; AICAr: 5-aminoimidazole-4-carboxamide ribonucleoside; AICAR: 5-aminoimidazole-4-carboxamide ribonucleotide; CMP: cytidine-5’-monophosphate; GMP: guanosine-5’-monophosphate; IMP: inosine-5’-monophosphate; OMP: orotidine-5’-monophosphate; PRPP: phosphoribosylpyrophosphate; SAICAr: succinyl-5-aminoimidazole-4-carboxamide-1-ribonucleoside; SAICAR: succinyl-5-aminoimidazole-4-carboxamide-1-ribonucleotide; S-AMP: adenylosuccinate; TMP: thymidine-5’-monophosphate; UMP: uridine-5’-monophosphate; XMP: xanthosine-5’-monophosphate.
Drawn using Inkscape 0.91pre4 (Open Source Software licensed under the GNU General Public License).
(TIF)

S1 Text Preparation of unlabeled standard and internal standard (IS) solutions, calibrators and Quality Controls (QCs). (DOC)

S2 Text Selected clinical data of the examined patients with known diagnosis (*n* = 10). (DOC)

S1 Table Extended details of method development. (DOC)

S2 Table Assay validation using calibrators (*n* = 10) and chromatographic resolution of critical analyte pairs with mass spectrometric interference. (DOC)

S3 Table Assay validation using the internal quality control samples (IQC) (*n* = 10). (DOC)

## Abbreviations

ADA: adenosine deaminase (deficiency)
ADN: adenine
ADS: adenosine
ADSL: adenylosuccinate lyase (deficiency)
AICAr: 5-aminoimidazole-4-carboxamide ribonucleoside
AICAR: 5-aminoimidazole-4-carboxamide ribonucleotide
ALLOP: allopurinol
APRT: adenine phosphoribosyltransferase (deficiency)
ATIC: AICAR transformylase/IMP cyclohydrolase (deficiency)
BUIB: beta-ureidoisobutyric acid
BUP: beta-ureidopropionic acid
DHA: 2,8-dihydroxyadenine
DHP: dihydropyrimidinase (deficiency)
DHT: 5,6-dihydrothymine
DHU: 5,6-dihydrouracil
dAdo: 2-deoxyadenosine
dGui: 2-deoxyguanosine
dIno: 2-deoxyinosine
dUrd: 2-deoxyuridine
DPD: dihydropyrimidine dehydrogenase (deficiency)
DPT: Diagnostic Proficiency Testing (ERNDIM)
EQC: ERNDIM external quality control
Guo: guanosine
HGPRT: hypoxanthine-guanine phosphoribosyltransferase (deficiency)
HMU: 5-hydroxymethyluracil
Hypo: hypoxanthine
Ino: inosine
IQC: internal quality control
MoCoD: molybdenum cofactor deficiency
Oro: orotic acid
Ord: orotidine
PNP: purine nucleoside phosphorylase (deficiency)
PRPPS: phosphoribosylpyrophosphate synthetase (superactivity)
PSU: pseudouridine
SAICAr: succinyl-5-aminoimidazole-4-carboxamide-1-ribonucleoside
SAICAR: succinyl-5-aminoimidazole-4-carboxamide-1-ribonucleotide
SAdo: succinyladenosine
THD: thymidine
Thy: thymine
TP: thymidine phosphorylase (deficiency)
UMPS: uridine monophosphate synthetase (deficiency)
UPB1: beta-ureidopropionase (deficiency)
URA: uracil
Xan: xanthine
XDH: xanthine dehydrogenase (deficiency)
