# Supplementary figures and images for: Extended diagnosis of purine and pyrimidine disorders from urine: LC MS/MS assay development and clinical validation
Source: PLoS One. 2019 Feb 28;14(2):e0212458. doi: 10.1371/journal.pone.0212458 (PMC6394934; doi:10.1371/journal.pone.0212458)

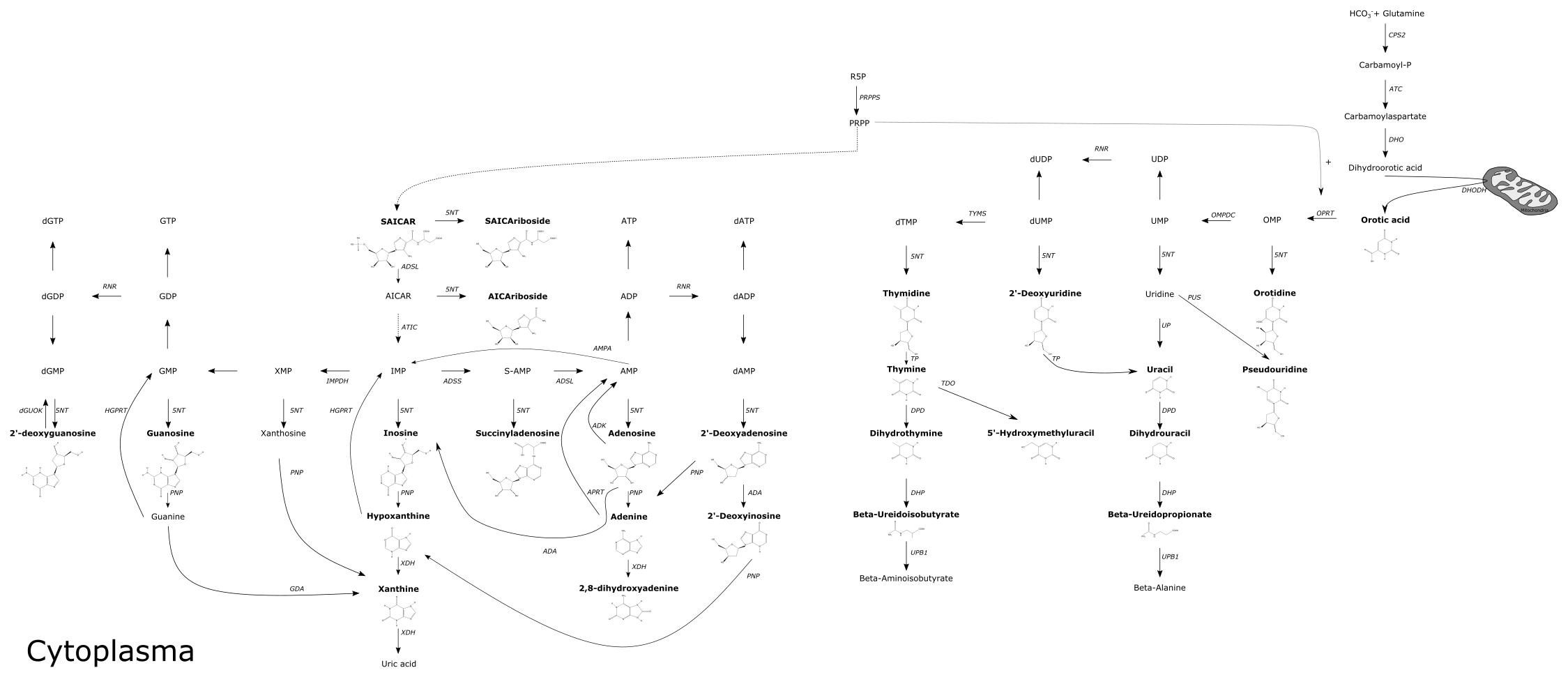

Supplement: S1 Fig — Metabolites detected by the presented method are printed in bold. Purines: Enzymes of the de novo purine synthesis: PRPPS: phosphoribosyl pyrophosphate synthetase; ADSL: adenylosuccinate lyase (adenylosuccinase); ATIC: AICAR transformylase/IMP cyclohydrolase; ADSS: adenylosuccinate synthetase. Enzymes of purine catabolism: AMPA: AMP deaminase; 5NT: 5'-nucleotidase(s); ADA: adenosine deaminase; PNP: purine nucleoside phosphorylase; XDH: xanthine dehydrogenase (xanthine oxidase); GDA: guanine deaminase; IMPDH: IMP dehydrogenase. Enzymes of purine salvage: HGPRT: hypoxanthine-guanine phosphoribosyltransferase; dGUOK: deoxyguanosine kinase; APRT: adenine phosphoribosyltransferase; ADK: adenosine kinase. Pyrimidines: Enzymes of the de novo pyrimidine synthesis: CPS2: carbamoylphosphate synthetase II: ATC: aspartate transcarbamoylase; DHO: dihydroorotase (CAD is comprised of CPS2, ATC and DHO); DHODH: dihydroorotate dehydrogenase; OPRT: orotate phosphoribosyltransferase; OMPDC: OMP decarboxylase (UMPS = uridine monophosphate synthetase is comprised of OPRT and OMPDC). Enzymes of pyrimidine catabolism: 5NT: 5'-nucleotidase(s); UP: uridine phosphorylase(s); TP: thymidine phosphorylase; DPD: dihydropyrimidine dehydrogenase; DHP: dihydropyrimidinase; UPB1: beta-ureidopropionase. Enzymes of pyrimidine salvage: PUS: pseudouridine synthase; TDO: thymine dioxygenase. Ribonucleotide reductase (RNR) and thymidylate synthetase (TYMS) are used in the synthesis of deoxynucleotides. AMP: adenosine-5’-monophosphate; AICAr: 5-aminoimidazole-4-carboxamide ribonucleoside; AICAR: 5-aminoimidazole-4-carboxamide ribonucleotide; CMP: cytidine-5’-monophosphate; GMP: guanosine-5’-monophosphate; IMP: inosine-5’-monophosphate; OMP: orotidine-5’-monophosphate; PRPP: phosphoribosylpyrophosphate; SAICAr: succinyl-5-aminoimidazole-4-carboxamide-1-ribonucleoside; SAICAR: succinyl-5-aminoimidazole-4-carboxamide-1-ribonucleotide; S-AMP: adenylosuccinate; TMP: thymidine-5’-monophosphate; UMP: uridine-5’-m [file pone.0212458.s001.tif]
