# Supplementary material for: Extended diagnosis of purine and pyrimidine disorders from urine: LC MS/MS assay development and clinical validation
Source: PLoS One. 2019 Feb 28;14(2):e0212458. doi: 10.1371/journal.pone.0212458 (PMC6394934; doi:10.1371/journal.pone.0212458)
Supplement: S1 Text — (DOC) [file pone.0212458.s002.doc]

**S1 Text**

**Manuscript title**

Extended diagnosis of purine and pyrimidine disorders from urine: LC‑MS/MS assay development and clinical validation

Péter Monostori^1*^, Glynis Klinke^1^, Jana Hauke^1^, Sylvia Richter^1^, Jörgen Bierau^2^, Sven F. Garbade^1^, Georg F. Hoffmann^1^, Claus-Dieter Langhans^1^, Dorothea Haas^1¶^, Jürgen G. Okun^1¶^

^1^ Department of General Pediatrics, Division of Neuropediatrics and Metabolic Medicine, Center for Pediatric and Adolescent Medicine, University Hospital Heidelberg, Heidelberg, Germany

^2^ Department of Clinical Genetics, Maastricht University Medical Center, Maastricht, The Netherlands

^¶^These authors contributed equally to this work.

*** Corresponding author**

E‑mail: monostoripeter@gmail.com (PM)

**S1 Text: Preparation of unlabeled standard and internal standard (IS) solutions, calibrators and Quality Controls (QCs)**

*Preparation of unlabeled standard stock solutions*

Stock solutions were prepared in 10 mM concentration each (exceptions: SAICAr 1 mM, succinyladenosine 2 mM) by dissolving unlabeled standards separately in solvents as follows:

| **Analyte** | **Solvent for stock solution** |
| --- | --- |
| Uracil | PUPYR stock solution buffer with 2% 1M NaOH |
| Dihydrouracil | PUPYR stock solution buffer |
| Thymine | PUPYR stock solution buffer |
| Dihydrothymine | PUPYR stock solution buffer |
| Beta-Ureidopropionic acid | PUPYR stock solution buffer |
| Adenine | PUPYR stock solution buffer |
| Hypoxanthine | water with 10% formic acid (99%) |
| Allopurinol | 1M NaOH |
| Beta-Ureidoisobutyric acid | PUPYR stock solution buffer |
| Xanthine | 1M NaOH |
| 2,8-Dihydroxyadenine | water with 5% 1M NaOH |
| Deoxyadenosine | PUPYR stock solution buffer |
| AICAr | PUPYR stock solution buffer |
| Adenosine | PUPYR stock solution buffer |
| 5-Hydroxymethyluracil | water |
| Orotic acid | water with 2% 1M NaOH |
| Deoxyuridine | PUPYR stock solution buffer |
| Thymidine | PUPYR stock solution buffer |
| Pseudouridine | PUPYR stock solution buffer |
| Deoxyinosine | PUPYR stock solution buffer |
| Deoxyguanosine | PUPYR stock solution buffer with 1% formic acid (99%) |
| Inosine | PUPYR stock solution buffer |
| Guanosine | water with 1% 1M NaOH |
| Orotidine | water |
| SAICAr (1 mM) | water |
| Succinyladenosine (2 mM) | methanol |

AICAr: 5‑aminoimidazole-4-carboxamide ribonucleoside; SAICAr: succinyl‑5‑aminoimidazole-4-carboxamide-1-ribonucleoside.

Please, note that the pH of the stock solutions and the resulting pH of the calibrators are critical, even if some substances may also dissolve in solvents other than stated here. Under alkaline conditions, dihydrouracil and dihydrothymine would degrade to beta-ureidopropionic acid and beta-ureidoisobutyric acid, respectively. Preparation of the PUPYR stock solution buffer is detailed in the next section.

Stock solutions of SAICAr, succinyladenosine and SAICAR were stored in aliquots at ‑80 °C. All other stock solutions were stored in aliquots at ‑20 °C.

*Preparation of the PUPYR stock solution buffer*

50 mM acetic acid adjusted to pH 4.0 with 25% ammonium hydroxide and then adjusted to pH 2.8 with 99% formic acid.

*Preparation of calibrators*

The highest level calibrator (Cal10) was prepared by spiking ultrapure water with stock solutions of unlabeled standards to obtain a 256 μM solution. Further calibrators (Cal09 to Cal02) were prepared via 2‑fold serial dilutions of the respective previous calibrator, mixed with equal volumes of water. Cal01 contained water without spiking. Accordingly, analyte levels in calibrators Cal01 to Cal10 were as follows: 0, 1, 2, 4, 8, 16, 32, 64, 128 and 256 μM. The aliquoted calibrators were then stored at ‑20 °C.

*Preparation of internal standard (IS) stock solutions*

Internal standard (IS) stock solutions were prepared in 10 mM concentration by dissolving labelled ISs separately in solvents as follows:

| **Internal standard** | **Solvent for stock solution** |
| --- | --- |
| Uracil-IS (1,3-^15^N_2_) | 50% isopropanol with 1% 1M NaOH |
| 5,6-Dihydrouracil-IS (^13^C_4_ ^15^N_2_) | 50% isopropanol |
| Thymine-IS (d_4_) | 50% isopropanol |
| 5,6-Dihydrothymine-IS (d_3_ + methyl-d_3_) | 50% isopropanol |
| Hypoxanthine-IS (^13^C_5_) | 50% isopropanol with 10% formic acid (99%) |
| Xanthine-IS (1,3-^15^N_2_) | 50% isopropanol with 2% 1M NaOH |
| Orotic Acid-IS (1,3-^15^N_2_) | water with 1% 1M NaOH |
| Thymidine-IS (^13^C_10_ ^15^N_2_) | 50% isopropanol |
| 2-Deoxyguanosine-IS (^13^C_10_ ^15^N_5_) | 50% isopropanol |
| Inosine-IS (^15^N_4_) | 50% isopropanol |
| Guanosine-IS (^15^N_5_) | water with 1% 1M NaOH |

Please, note that the pH of the IS stock solutions and the resulting pH of the IS working solution are critical. Please, refer to the note in section „Preparation of unlabeled standard stock solutions”.

*Preparation of IS working solution*

The IS working solution was prepared by spiking ultrapure water with IS stock solutions to obtain a 25 μM solution.

The IS stock solutions and the working solution were stored in aliquots at ‑20 °C.

*Preparation of quality controls (QCs)*

For the preparation of the external QC (EQC), the lyophilized „Control Purines and Pyrimidines” material from ERNDIM (supplier: MCA Laboratory, Queen Beatrix Hospital, Winterswijk, The Netherlands) was first reconstituted with water according to the provider’s instructions (5.0 ml water to 1 vial), and then further diluted with water to reach a creatinine level of 1.0 mM. The analyte levels of the actual charge of EQC used during method development (LOT 2015.010) were provided in the respective certificate and are detailed in the following table:

| **Analyte** | **Concentration (μM)** |
| --- | --- |
| Uracil | 21.7 |
| Dihydrouracil | 19.7 |
| Thymine | 11.9 |
| Dihydrothymine | 21.3 |
| Adenine | 8.5 |
| Hypoxanthine | 35.0 |
| Xanthine | 29.0 |
| Deoxyadenosine | 11.5 |
| AICAr | 15.9 |
| Adenosine | 10.5 |
| 5-Hydroxymethyluracil | 15.0 |
| Orotic acid | 24.2 |
| Deoxyuridine | 13.0 |
| Thymidine | 6.9 |
| Pseudouridine | 27.3 |
| Deoxyinosine | 11.6 |
| Deoxyguanosine | 11.5 |
| Inosine | 15.1 |
| Guanosine | 17.0 |
| Orotidine | 4.2 |

AICAr: 5‑aminoimidazole-4-carboxamide ribonucleoside.

The internal QC (IQC) was prepared by mixing stock solutions of unlabeled standards and diluting with ultrapure water to obtain a 20 μM solution. The IQC contained all metabolites of the EQC plus beta‑ureidopropionic acid, allopurinol, beta‑ureidoisobutyric acid, 2,8‑dihydroxyadenine and succinyladenosine. Both QCs were stored similarly to the calibrators.
