# Supplementary material for: Extended diagnosis of purine and pyrimidine disorders from urine: LC MS/MS assay development and clinical validation
Source: PLoS One. 2019 Feb 28;14(2):e0212458. doi: 10.1371/journal.pone.0212458 (PMC6394934; doi:10.1371/journal.pone.0212458)
Supplement: S2 Text — (DOC) [file pone.0212458.s003.doc]

**S2 Text**

**Manuscript title**

Extended diagnosis of purine and pyrimidine disorders from urine: LC‑MS/MS assay development and clinical validation

Péter Monostori^1*^, Glynis Klinke^1^, Jana Hauke^1^, Sylvia Richter^1^, Jörgen Bierau^2^, Sven F. Garbade^1^, Georg F. Hoffmann^1^, Claus-Dieter Langhans^1^, Dorothea Haas^1¶^, Jürgen G. Okun^1¶^

^1^ Department of General Pediatrics, Division of Neuropediatrics and Metabolic Medicine, Center for Pediatric and Adolescent Medicine, University Hospital Heidelberg, Heidelberg, Germany

^2^ Department of Clinical Genetics, Maastricht University Medical Center, Maastricht, The Netherlands

^¶^These authors contributed equally to this work.

*** Corresponding author**

E‑mail: monostoripeter@gmail.com (PM)

**S2 Text: Selected clinical data of the examined patients with known diagnosis (*n*=10). LC‑MS/MS data are presented in Fig 2 of the main Manuscript.**

P01: 8‑month‑old female, microcephaly, psychomotor developmental delay, consanguineous parents

P02: 31‑year‑old female, global developmental delay, pathological eye movements, diagnosis confirmed by molecular analysis

P03: 16‑year‑old male, epilepsy, normal cognitive development, no megaloblastic anemia, diagnosed at the age of 2 years, confirmed by molecular analysis, under uridine therapy. Published in: *Grohmann et al.: Hereditary orotic aciduria with epilepsy and without megaloblastic anemia. Neuropediatrics 2015; 46: 123-125.*

P04: 1‑month‑old female, muscular hypotonia, difficulties in swallowing, diminished reflexes, consanguineous parents

P05: 12‑year‑old male, autoaggressive behavior, generalized dystonia, cognitive impairment, diagnosis confirmed by molecular analysis

P06: 4.5‑year‑old male, delay in speech development, aggressive behavior

P07: 3.5‑year‑old female, no clinical data available

P08: 5.5‑year‑old female, no clinical data available

P09: 1‑year‑old female, psychomotor delay, myoclonus, muscular hypotonia, nystagmus, diagnosis confirmed by molecular analysis

P10: 8‑month‑old male, no clinical data available
