# Supplementary material for: Extended diagnosis of purine and pyrimidine disorders from urine: LC MS/MS assay development and clinical validation
Source: PLoS One. 2019 Feb 28;14(2):e0212458. doi: 10.1371/journal.pone.0212458 (PMC6394934; doi:10.1371/journal.pone.0212458)
Supplement: S1 Table — (DOC) [file pone.0212458.s004.doc]

**S1 Table**

**Manuscript title**

Extended diagnosis of purine and pyrimidine disorders from urine: LC‑MS/MS assay development and clinical validation

Péter Monostori^1*^, Glynis Klinke^1^, Jana Hauke^1^, Sylvia Richter^1^, Jörgen Bierau^2^, Sven F. Garbade^1^, Georg F. Hoffmann^1^, Claus-Dieter Langhans^1^, Dorothea Haas^1¶^, Jürgen G. Okun^1¶^

^1^ Department of General Pediatrics, Division of Neuropediatrics and Metabolic Medicine, Center for Pediatric and Adolescent Medicine, University Hospital Heidelberg, Heidelberg, Germany

^2^ Department of Clinical Genetics, Maastricht University Medical Center, Maastricht, The Netherlands

^¶^These authors contributed equally to this work.

*** Corresponding author**

E‑mail: monostoripeter@gmail.com (PM)

**S1 Table: Extended details of method development.**

This table is intended to share our experience obtained during method development, as well as reasons why a given configuration was selected for subsequent method validation.

| **Assay parameter** | **Configurations tested** | **Notes** |
| --- | --- | --- |
| **Column** | ACE Excel C18‑AR 100×3.0mm; 1.7 μm  Phenomenex Kinetex Polar C18 150×2.1 mm,2.6 μm  Phenomenex Kinetex Biphenyl 150×2.1 mm, 2.6 μm | Test columns were selected on the basis of literature data and analyte properties. The ACE Excel C18‑AR 100×3.0mm; 1.7 μm column provided best resolution, peak shape and signal intensity among the tested columns. |
| **Eluents** | A: 20 mM Amm. formate + 20 mM formic acid  A. 50 mM acetic acid, adjusted to pH 4.0 with NH_4_OH, adjusted to pH 2.8 with formic acid  A: Water +0.4% formic acid  B: MeOH/water 50/50 (v/v)  B: ACN/MeOH 50/50 (v/v)  B: MeOH / 20 mM Amm. formate + 20 mM formic acid 50/50 (v/v) | Resolution and peak shape was most optimal with water +0.4% formic acid as Eluent A and MeOH/water 50/50 (v/v) as Eluent B. |
| **Sample preparation format** | 96‑well Merck MultiScreen filter plate + collection plate (volume of both: 2 ml)  Merck Ultrafree‑MC VV 0.1 µm centrifugal filter units | There were no significant differences between analytical results for the two sample preparation formats. However, filter plates offered higher throughput, less opportunity for technical errors and lower cost. |

Underlined configurations were selected for subsequent method validation.

ACN: acetonitrile; MeOH: methanol.
