# Supplementary material for: Extended diagnosis of purine and pyrimidine disorders from urine: LC MS/MS assay development and clinical validation
Source: PLoS One. 2019 Feb 28;14(2):e0212458. doi: 10.1371/journal.pone.0212458 (PMC6394934; doi:10.1371/journal.pone.0212458)
Supplement: S2 Table — (DOC) [file pone.0212458.s005.doc]

**S2 Table**

**Manuscript title**

Extended diagnosis of purine and pyrimidine disorders from urine: LC‑MS/MS assay development and clinical validation

Péter Monostori^1*^, Glynis Klinke^1^, Jana Hauke^1^, Sylvia Richter^1^, Jörgen Bierau^2^, Sven F. Garbade^1^, Georg F. Hoffmann^1^, Claus-Dieter Langhans^1^, Dorothea Haas^1¶^, Jürgen G. Okun^1¶^

^1^ Department of General Pediatrics, Division of Neuropediatrics and Metabolic Medicine, Center for Pediatric and Adolescent Medicine, University Hospital Heidelberg, Heidelberg, Germany

^2^ Department of Clinical Genetics, Maastricht University Medical Center, Maastricht, The Netherlands

^¶^These authors contributed equally to this work.

*** Corresponding author**

E‑mail: monostoripeter@gmail.com (PM)

**S2 Table: Assay validation using calibrators (*n*=10) and chromatographic resolution of critical analyte pairs with mass spectrometric interference.**

| **Analyte** | **Linearity (R^2^)** | **LOD (μM)** | **LLOQ (μM)** | **ULOQ (μM)** | **Retention time (min)** |
| --- | --- | --- | --- | --- | --- |
| Uracil | 0.9995 | 0.25 | 0.5 | >250 | 3.54±0.01 |
| Dihydrouracil | 0.9989 | 0.5 | 1.25 | >250 | 3.57±0.01 |
| Thymine | 0.9993 | 0.125 | 0.25 | 250 | 5.62±0.02 |
| Dihydrothymine | 0.9986 | 0.25 | 0.5 | 250 | 5.60±0.02 |
| Beta-Ureidopropionic acid | 0.9990 | 0.5 | 1.25 | 125 | 3.33±0.01 |
| Adenine | 0.9907 | 0.025 | 0.05 | 125 | 3.44±0.02 |
| Hypoxanthine | 0.9996 | 0.05 | 0.125 | 125 | 4.11±0.03 |
| Allopurinol | 0.9979 | 0.125 | 0.25 | 250 | 5.41±0.03 |
| Beta-Ureidoisobutyric acid | 0.9990 | 0.5 | 1.25 | 125 | 4.98±0.02 |
| Xanthine | 0.9990 | 0.025 | 0.05 | 125 | 4.80±0.03 |
| 2,8‑Dihydroxyadenine | 0.9901 | 0.05 | 0.125 | 125 | 3.41±0.01 |
| Deoxyadenosine | 0.9905 | 0.005 | 0.0125 | 50 | 5.58±0.02 |
| AICAr | 0.9993 | 0.0125 | 0.025 | 50 | 4.48±0.03 |
| Adenosine | 0.9941 | 0.005 | 0.0125 | 50 | 5.32±0.03 |
| 5‑Hydroxymethyluracil | 0.9979 | 1.25 | 2.5 | 125 | 3.48±0.01 |
| Orotic acid | 0.9990 | 0.5 | 1.25 | 250 | 3.30±0.05 |
| Deoxyuridine | 0.9984 | 0.5 | 1.25 | 250 | 5.46±0.02 |
| Thymidine | 0.9971 | 1.25 | 2.5 | 125 | 6.67±0.02 |
| Pseudouridine | 0.9974 | 0.5 | 1.25 | 250 | 3.37±0.02 |
| Deoxyinosine | 0.9991 | 0.025 | 0.05 | 250 | 6.16±0.02 |
| Deoxyguanosine | 0.9988 | 0.0125 | 0.025 | 250 | 6.03±0.03 |
| Inosine | 0.9993 | 0.025 | 0.05 | 250 | 5.84±0.03 |
| Guanosine | 0.9990 | 0.025 | 0.05 | 250 | 5.79±0.03 |
| Orotidine | 0.9971 | 0.5 | 1.25 | 250 | 2.66±0.03 |
| SAICAr | 0.9984 | 0.25 | 0.5 | 250 | 5.99±0.02 |
| Succinyladenosine | 0.9970 | 0.05 | 0.125 | 250 | 7.70±0.03 |

AICAr: 5‑aminoimidazole-4-carboxamide ribonucleoside; SAICAr: succinyl‑5‑aminoimidazole-4-carboxamide-1-ribonucleoside; R^2^: coefficient of determination; LOD: limit of detection; LLOQ: lower limit of quantitation; ULOQ: upper limit of quantitation.

Chromatographic resolution of critical analyte pairs with mass spectrometric interference.

| **Analyte 1** | **Analyte 2** | ***Rs*** |
| --- | --- | --- |
| Adenosine-IS parent | Inosine parent | 3.41 |
| Orotidine parent | Guanosine-IS parent | 21.80 |
| Adenosine parent | Deoxyguanosine parent | 4.41 |
| Adenine parent | Hypoxanthine parent | 4.04 |
| Adenine parent | Allopurinol parent | 12.85 |
| Deoxyinosine daughter | Adenine parent | 17.77 |
| Hypoxanthine parent | Allopurinol parent | 8.14 |
| Hypoxanthine parent | Deoxyinosine parent | 12.79 |
| Deoxyinosine daughter | Allopurinol parent | 4.92 |
| Beta-Ureidopropionic acid daughter | Dihydrouracil parent | 2.05 |
| Adenosine daughter | Adenine parent | 11.61 |
| Thymidine daughter | Thymine parent | 7.82 |
| Guanosine-IS daughter | Orotic acid parent | 17.49 |
| Orotidine daughter | Orotic acid parent | 4.06 |
| Beta-Ureidoisobutyric acid daughter | Dihydrothymine parent | 3.66 |
| Succinyladenosine daughter | Deoxyadenosine parent | 13.90 |
| Succinyladenosine daughter | Adenosine parent | 14.78 |
| Orotic acid daughter | Uracil parent | 2.01 |
| AICAr daughter | Thymine parent | 7.08 |

IS: internal standard; AICAr: 5‑aminoimidazole-4-carboxamide-ribonucleoside.

Resolution (*R_s_*) was calculated via the following equation: *R_s_=1.18(t_2_‑t_1_)/(w_0.5,1_+w_0.5,2_)* where *t_1_* and *t_2_* are the retention times of the respective peaks and *w_0.5,1_* and *w_0.5,2_* are the peak widths at half peak height. Baseline resolution (*R_s_*≥1.5) was achieved for all critical pairs.
