# Supplementary material for: Extended diagnosis of purine and pyrimidine disorders from urine: LC MS/MS assay development and clinical validation
Source: PLoS One. 2019 Feb 28;14(2):e0212458. doi: 10.1371/journal.pone.0212458 (PMC6394934; doi:10.1371/journal.pone.0212458)
Supplement: S3 Table — (DOC) [file pone.0212458.s006.doc]

**S3 Table**

**Manuscript title**

Extended diagnosis of purine and pyrimidine disorders from urine: LC‑MS/MS assay development and clinical validation

Péter Monostori^1*^, Glynis Klinke^1^, Jana Hauke^1^, Sylvia Richter^1^, Jörgen Bierau^2^, Sven F. Garbade^1^, Georg F. Hoffmann^1^, Claus-Dieter Langhans^1^, Dorothea Haas^1¶^, Jürgen G. Okun^1¶^

^1^ Department of General Pediatrics, Division of Neuropediatrics and Metabolic Medicine, Center for Pediatric and Adolescent Medicine, University Hospital Heidelberg, Heidelberg, Germany

^2^ Department of Clinical Genetics, Maastricht University Medical Center, Maastricht, The Netherlands

^¶^These authors contributed equally to this work.

*** Corresponding author**

E‑mail: monostoripeter@gmail.com (PM)

**S3 Table: Assay validation using the internal quality control samples (IQC) (*n*=10).**

| **Analyte** | **Analyte level (μM)** | **CV (%) interday** | **CV (%) intraday** | **CV (%) between injections of a single sample extract** | **Recovery (%)** |
| --- | --- | --- | --- | --- | --- |
| Uracil | 20 | 4.5 | 2.6 | 3.0 | 99.2 |
| Dihydrouracil | 20 | 4.7 | 4.3 | 4.0 | 96.2 |
| Thymine | 20 | 3.8 | 4.8 | 3.4 | 99.2 |
| Dihydrothymine | 20 | 3.9 | 3.5 | 2.5 | 103.8 |
| Beta-Ureidopropionic acid | 20 | 4.3 | 3.3 | 2.9 | 95.9 |
| Adenine | 20 | 5.7 | 3.6 | 2.0 | 110.4 |
| Hypoxanthine | 20 | 3.7 | 4.0 | 2.8 | 98.6 |
| Allopurinol | 20 | 3.3 | 3.6 | 2.8 | 105.6 |
| Beta-Ureidoisobutyric acid | 20 | 4.4 | 5.7 | 3.4 | 93.0 |
| Xanthine | 20 | 5.3 | 5.7 | 2.6 | 100.5 |
| 2,8‑Dihydroxyadenine | 20 | 7.2 | 6.6 | 6.7 | 108.9 |
| Deoxyadenosine | 20 | 6.3 | 4.2 | 2.9 | 109.6 |
| AICAr | 20 | 4.4 | 3.9 | 4.4 | 98.5 |
| Adenosine | 20 | 5.6 | 4.6 | 3.5 | 107.8 |
| 5‑Hydroxymethyluracil | 20 | 10.7 | 7.9 | 3.7 | 102.1 |
| Orotic acid | 20 | 7.9 | 4.0 | 4.8 | 99.0 |
| Deoxyuridine | 20 | 4.8 | 4.8 | 5.2 | 91.1 |
| Thymidine | 20 | 11.5 | 5.7 | 9.6 | 94.3 |
| Pseudouridine | 20 | 3.3 | 2.1 | 2.8 | 104.3 |
| Deoxyinosine | 20 | 3.3 | 3.4 | 2.7 | 94.4 |
| Deoxyguanosine | 20 | 5.8 | 5.0 | 3.5 | 96.9 |
| Inosine | 20 | 2.8 | 2.9 | 3.3 | 99.0 |
| Guanosine | 20 | 8.8 | 6.1 | 2.4 | 94.8 |
| Orotidine | 20 | 5.9 | 3.2 | 5.7 | 107.0 |
| Succinyladenosine | 20 | 2.8 | 2.8 | 1.9 | 84.6 |

CV: coefficient of variation; AICAr: 5‑aminoimidazole-4-carboxamide ribonucleoside.
